# Clinical Studies in Vice and in Insanity

**Published:** 1900-09

**Authors:** 


**Clinical Studies in Vice and in Insanity.** By GEORGE R. WILSON, M.D. Pp. xi., 234. Edinburgh: William F. Clay. 1899.

This book contains a number of well narrated clinical records, by the aid of which types of alcoholists, the alcoholic diathesis, and certain phases of insanity are studied; and which constitute a basis for careful and original reflections upon various subjects, clinical, pathological and therapeutical arising in connection therewith. In the preface, Dr. Wilson discusses some questions relative to alcoholism, the simplification of its nomenclature, the true character of the early mental stages, the nature and mode of progression of the alcoholic lesion, and the therapeutics. A brief account of the dendritic system of the cortex and of the dynamics of brain-function, is followed by the author's views on the pathology of alcoholic degeneration. He considers this to commence in a state of diminished excitability of the dendritic system, probably of trophic nature, and to be followed by all the effects that result from impaired conduction and activity in the higher centres (as positive lesions) and consequent excess in function of lower mechanisms (as negative lesions). At first, fresh mental constructions are limited and initiative fails; later on already organised tracts succumb, and the function of discipline is impaired. Hence the most obvious and early of the effects of alcoholism is loss of purposive ability. The author discusses at length what he terms the tonic circulation of nerve force, and the essential part played in its maintenance by the dendritic layers. To the depreciation in this process he ascribes all the psychic symptoms of alcoholism; as negative and primary, the impairment proceeding to extinction of initiative, amnesia, anæsthesia, failure of corrective judgment; as positive and secondary, emotional manifestations, hyperæsthesias and false perceptions. His theories are applied in detail in the studies of aphasia and hallucination. The clinical records are instructive and interesting reading, and frequently interspersed will be found suggestive digressions, which evidence the thoughtful spirit in which the book has been conceived: there is also shewn earnest desire to grapple with the difficulties in the treatment of the insane class. Particularly commendable are the author's remarks concerning the need for the study of the patient's subjective state in a sympathetic spirit, and the conscientious avowal of his belief in the efficacy of mechanical restraint in certain cases: we cordially endorse his views. This work will well repay careful perusal. The publishers have produced it carefully.

